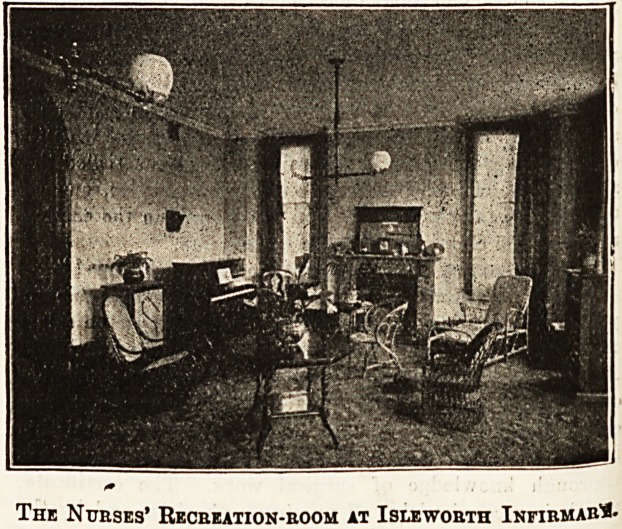# "The Hospital" Nursing Section

**Published:** 1906-06-30

**Authors:** 


### The Hospital.

### Nursing Section.

Contributions for "The Hospital," should be addressed to the Editor, "The Hospital" Nursing Section, 28 & 29 Southampton Street, Strand, London, W.C.

No. 1,032.—Vol. XL. SATURDAY, JUNE 30, 1906.

### Notes on News from the Nursing World.

### LADY MINTO AND ENGLISH NURSES FOR INDIA.

We are glad to learn that the Countess of Minto has fulfilled the expectations we expressed last year, and is following in the footsteps of Lady Curzon. She has warmly interested herself in a movement for the organisation of a staff of English trained nurses for the European community in India. The scheme, which the wife of the Viceroy supports, embraces Northern India, Beluchistan, and Burma, and is approved by the Anglo-Indian Press. The Viceroy himself has given 5,800 rupees, and the contributories include 1,500 rupees from Lord Kitchener.

### QUEEN VICTORIA'S JUBILEE INSTITUTE.

The half-yearly Conference of members of the Council of Queen Victoria's Jubilee Institute for Nurses, and of representatives of its affiliated district nursing associations in the metropolitan area, was held on Wednesday last week, Sir William Cameron Gull in the chair. Thirty-three representatives of district nursing associations were present. We understand that various matters in connection with the organisation and work of the nursing associations were discussed, including the best methods of raising funds, the question of payments by patients for services rendered, and the provision of nurses for infectious cases. The practice in the various districts with regard to these points was reported by the representatives, much valuable and helpful information being obtained.

### NURSING IN VOLUNTEER MEDICAL HOSPITALS.

It is intimated in the *British Medical Journal* that a number of nurses are needed, or will be needed, for service in Volunteer medical hospitals. The institutions which are to be erected under the auspices of the Volunteer Medical Service are already, it is stated, assured of a sufficient number of Volunteer medical officers to provide surgeons, "but there is no machinery for the provision of nurses." This difficulty, we do not doubt, will be overcome. There may not be a large number of nurses in a position to offer themselves as volunteers, but we think that as many as are required will make themselves available for the scheme of national defence as soon as the want of their assistance is brought home to them. What is the use of nurses volunteering their help when there are no patients to attend?

### PROBATIONERS IN THEIR TEENS.

The Swindon and Highworth Guardians have decided to ask the Local Government Board to allow them to reduce the minimum age limit of probationers from 21 to 19. Their decision is due to difficulties in obtaining probationers of the age required under the existing regulations. Some people think that it would be better to raise the minimum age for probationers employed in Poor-law infirmaries, and bring them in this respect into line with the hospitals. To reduce it would certainly be retrogressive policy, which would not tend to render the service more attractive to the class most likely to be of value. We admit that now and again a girl of nineteen is not unfit to be entrusted with the work. But in the vast majority of cases this is not so, and there is a consensus of opinion among recognised authorities in the nursing world that the duty of attending the sick cannot, as a rule, be performed satisfactorily except by women who have had some experience of the world, are acquainted with its vicissitudes, and are readily amenable to the discipline which is essential in the wards. There is no rule which is not a hardship to the few, but we cannot give priority either to the interests of young women who are unable to wait until they are 21 years old before they seek some kind of employment, or to the needs of the Swindon and Highworth Guardians, in preference to the interests of nursing and nurses in general. The Local Government Board, we are convinced, will consider it requisite to refuse to flood the institutions under their control with probationers in their teens.

### THE NEW MATERNITY DEPARTMENT AT THE LONDON HOSPITAL.

The recent opening of the new maternity department at the London Hospital, having three sisters recognised as teachers by the Central Midwives Board, is an important development made possible by modern methods in treatment. The organisation of this department is completed, and its official recognition as a training school for midwives may be expected shortly. It has led to the endowment of a fund to provide milk for poor mothers in the district round the hospital, and to the discovery that few of the in-patients have any clothes for the new babies, which has caused Lady Derby to supply each year one hundred bundles of suitable things in the way of baby clothes to be given to the mothers by Sister Marie Celeste. The tendency nowadays is perhaps to do too much rather than too little for hospital patients, and care will have to be taken that Lady Derby's generosity is not abused.

### NURSING AT A MODERN POOR-LAW INFIRMARY.

In the course of the interesting story of the evolution of a Poor-law Training School, told by the matron of Isleworth Infirmary to our Commissioner, which appears in our columns to-day, Miss Moriarty was asked if she thought that probationers trained in Poor-law schools are particularly well-qualified for private nursing. Bearing in mind that she herself was trained at St. Thomas's Hospital, her answer is worth noting. She is satisfied that they are "better suited for private work than hospital-trained nurses"; and as one of her reasons she says that as probationers "they are obliged to be sympathetic and kindly to individual patients." If, however, probationers in hospitals, are not sympathetic and kindly to individual patients, they have a very curious sense of duty, and are strangely unconscious of the true nature of their work. We fully endorse the opinion of the matron of Isleworth Infirmary that there is an excellent opening for well-educated girls to do useful work in Poor-law infirmaries where the higher posts are open to them, and the conditions are similar to those prevailing under the auspices of the Brentford Guardians; and we are heartily at one with her in her belief in nurses who regard nursing as a vocation, and do not look at it from the "case" point of view.

### A THEATRE SISTER FOR GRIMSBY.

Prior to the advent of the new matron at Grimsby Hospital the work of the theatre has been divided amongst the ward sisters. Miss Davies finding that, under this system, they were too often absent from the work, suggested the appointment of a theatre sister. The question came before the committee at their last meeting, and the Chairman strongly recommended that the proposal should be adopted. The cost, he stated, would be only £5 more, and the drawback that the services of one of the staff nurses would have to be dispensed with. It was agreed to appoint a sister-in-charge of the theatre, and one more progressive step has thus been made under the auspices of an enlightened authority.

### ENTERIC PATIENTS WITHOUT NURSES.

At the meeting of the Metropolitan Asylums Board on Saturday, the serious outbreak of enteric fever at Belmont Asylum was discussed. The Chairman of the Asylum Sub-Committee, who have been accused of delay in their efforts to cope with the outbreak, defended the action of himself and his colleagues. He stated that on May 14 two cases of fever, supposed to be enteric, occurred at Belmont Asylum. They were carefully watched, and after a time any doubts as to the nature of the disease became impossible. At the end of three weeks the number of cases had risen to 45, including two of the attendants. Two male trained nurses were then engaged, this assistance being afforded by the committee directly it was desired by the medical officer. This may have been so, but we agree with Mr. Elliott that trained nurses ought to have been engaged before the expiration of 21 days if the malady had previously been pronounced to be typhoid by the medical officer, and that when the necessity for employing them was tardily recognised, a staff, and not a couple, should have been engaged. The matter has been referred to the General Purposes Committee for investigation and report, but nothing can alter the fact that though in enteric fever nursing is of the utmost importance, the steadily increasing number of patients at Belmont were left without adequate nursing for three weeks. Such a state of things should be next to impossible in any of the institutions administered by the Metropolitan Asylums Board.

### FIVE NURSES IN ONE BEDROOM.

The sleeping accommodation provided for the nursing staff attached to Fulwood Workhouse, Lancashire, is sufficiently condemned by the statement of one of the Preston Guardians, who says that there are five nurses in one bedroom. The statement was made at the last meeting of the Guardians, when a report of the sub-committee in favour of the erection of a nurses' home was discussed. According to the same Guardian, the nurses are at present worse housed than in any workhouse in the country, and though several attempts were made to convict him of the use of exaggerated language, his statement about the five in one room was not disputed. The only extenuation urged was that the room is very large, and would accommodate 12 beds. Moreover, under existing arrangements, the nurses at Fulwood sleep, as another Guardian observed, with the noise of the institution ringing in their ears. In any case, we hope that the special committee, to whom the question was eventually referred, will strongly advise the Guardians to take action at once. We recently commented upon the case of three nurses sleeping in one room at a Poor-law Infirmary in the South of England, but the conditions of affairs at Fulwood Workhouse is still more objectionable. As to the plea of size, we suppose that even those who adopt it are not prepared to contend that nurses who work hard all day should sleep in a kind of ward at night. It is not surprising that in these circumstances there has admittedly been a difficulty in retaining the services of the staff.

### A SCANDAL AT EAST HAM.

Events have recently come to light at East Ham Convalescent Home which have resulted in the resignations of the matron and night nurse being accepted. At the last meeting of the East Ham Town Council Mr. Osborne, one of the members, said that the institution was a disgrace to the borough, and the sooner the building was closed the better. "Members of the staff had been seen going into the Home at half-past two in the morning in a state of intoxication." He stigmatised the Home as not a fit place to be sent to, and added that "one nurse, who was about to become a mother, was now in the workhouse infirmary, a burden on the rates." The matron has since denied any knowledge of the proceedings described by the Councillor, and stated that when the nurses had their night's leave she did not remain up to see them return, as that was the duty of the night nurse, who booked their time. The matron affirms that her resignation was sent in before the meeting of the Council took place.

### IMPERIAL MILITARY NURSING SERVICE.

We are officially informed that Miss H. C. Winzer, Miss M. A. Cachemaille, and Miss F. J. Mitchell have been appointed staff nurses in Queen

Alexandra's Imperial Military Nursing Service. Miss M. E. Richardson, sister, has been transferred to the Military Hospital, Hounslow, from the Cambridge Hospital, Aldershot; Miss E. L. McAllister, sister, to the Queen Alexandra Military Hospital, Millbank, from the Royal Victoria Hospital, Netley; Miss M. Steenson, sister, to the Military Hospital Portsmouth, on her return from South Africa; Miss A. S. Bond, R.R.C., sister, to the Military Hospital, Devonport, from the Military Hospital, Canterbury; Miss J. Hoadley, R.R.C., sister, to the Military Hospital, Canterbury, from the Cambridge Hospital, Aldershot; and Miss E. M. Fairchild, sister, to Cambridge Hospital, Aldershot, from the Royal Victoria Hospital, Netley. Miss C. T. Bilton, staff nurse, has been transferred to the Royal Victoria Hospital, Netley, from the Military Hospital, Hounslow; Miss G. M. Allen, staff nurse, to the Military Hospital, Colchester, from the Military Hospital, Portsmouth; Miss D. J. Saunder, staff nurse, to the Royal Victoria Hospital, Netley, from the Royal Arsenal Hospital, Woolwich; Miss A. M. S. Clapp, staff nurse, to the Royal Victoria Hospital, Netley, on appointment; Miss M. H. Congleton and Miss C. H. E. Gerahty, staff nurses, to the Military Hospital, Portsmouth, on appointment. The appointments of Miss C. W. Jones and Miss M. Plaskitt as staff nurses have been confirmed. Miss F. A. Dawson, Miss B. F. Perkins, Miss E. M. Lyde, Miss E. M. Perkins, Miss E. L. McAllister, and Miss G. M. Smith, staff nurses, have been promoted to be sisters.

### NEWCASTLE NURSES AND THE PENSION FUND.

At the Royal Infirmary, Newcastle-on-Tyne, a meeting was held on Wednesday evening last week of nurses and their friends in order to hear an address from Mr. Louis H. M. Dick, on the benefits and rules of the Royal National Pension Fund. Sir George Philipson presided, and in addition to Dr. Ranken Lyle, Mr. Orde, House Governor, and Mr. T. Oliver, Secretary, there were present Miss Aston, matron; Miss Clarke, superintendent nurse of the Union Hospital; Miss Houston, of the Dene Home Hospital; Miss Coleman, of the Cathedral Nursing Society; Miss Drummond, of the Northern Counties Home; Miss Hawthorn, of the Samaritan Hospital; Miss Ogston, of the City Hospital; and Miss Schwappe, of the Isolation Hospital, Wallsend. The Chairman having strongly commended the objects of the Pension Fund, Mr. Dick delivered his address, and a vote of thanks to him was subsequently proposed by Dr. Drummond, seconded by Sir Riley Lord. Subsequently, Sir George Philipson invited the audience to partake of refreshments provided by the matron and her staff, and the Pension Fund was discussed for a long time after the formal part of the meeting had terminated.

### THE VICTIMS OF THE HIGHGATE TRAM ACCIDENT.

No patients were received at the Holborn Infirmary in connection with the disastrous tram accident at Highgate on Saturday, but the whole of the injured were speedily removed to the Great Northern Central Hospital, where Miss Pearce, the matron, at the first warning news, gathered her staff together in order to meet the emergency. It was a terrible scene as one after another was brought in, some severely crushed and battered, others, suffering from concussion, apparently dead, and many seriously cut by the showers of broken glass from the wrecked shops. Everyone lent a willing hand, the matron herself assisting as well as directing. The crowd of anxious friends and relatives, hurrying to ascertain the safety of their own travellers, of course increased the confusion and difficulty, but order was restored at last. Most of the sufferers were able to return to their homes after having their injuries seen to and treated. Four only remained in the hospital on Monday, two of whom are still in a dangerous condition.

### INCREASE OF THE STAFF AT KINGSTON INFIRMARY.

At a meeting of the Kingston Guardians on Tuesday last, a resolution was passed increasing the nursing staff by seven—namely, three staff nurses and four probationers. This makes a total staff at the Kingston Poor-law Infirmary of 12 sisters and 40 probationers.

### A BADGE FOR MIDWIVES.

The increase in the number of midwives trained by the Association for Promoting the Training and Supply of Midwives has suggested to the Committee the advantage of giving their midwives a distinctive badge. Accordingly, a few simple rules have been drawn up by which, after a definite period of satisfactory work as district midwives among the poor, those trained under the auspices of the organisation become "Associates" and receive a badge, to be returned if they cease to do the special work in which the Association is interested. The first annual gathering of midwives belonging to the Association will be held, by invitation of Mrs. Wallace Bruce, Chairman of the Executive Committee, at 9 Airlie Gardens, Campden Hill, on Wednesday next, July 4, at 4 o'clock, when the badges will be presented by Lady Balfour of Burleigh.

### SHORT ITEMS.

On Saturday a man was sentenced at the West London Police Court to three months' hard labour for robbery at the Marylebone Infirmary. One of the nurses was aroused from sleep by a noise in her bedroom, and saw a man at her toilet table. She gave the alarm, and he was captured. His offence was aggravated by the fact that he had several times been an inmate of the institution.—With reference to our account of training midwives in Monmouthshire last week, we understand that there was not a single death in the practice under Sister Barrett's superintendence during the year.—The fourth Clapham open Examination for Midwifery took place on June 16. Eighteen candidates submitted themselves to written and oral examinations, and also to a clinical examination of cases in the wards of the Clapham Maternity Hospital. Only three candidates failed, and five took a place in the honours list with above five-eights of the maximum number of marks.

### The Nursing Outlook.

"From magnanimity, all fears above;
From nobler recompense, above applause,
Which owes to man's short outlook all its charm."

### A GREAT NURSE-TRAINING SCHOOL.

### III.—INCREASED COMFORT WITH EFFICIENCY.

HAVING dealt with the developments of the school, it remains for us to consider what has been done to increase the comfort and happiness of the individual nurse. With the opening of the Eva Lückes Home, well named in recognition of the matron's twenty-five years' service at the London Hospital, a total of 433 bedrooms for nurses, exclusive of 82 bedrooms and bed-sitting-rooms occupied by sisters, is now provided for the nursing staff. The provision of a separate bedroom for each nurse has produced the effect of making every room more attractive than before, because each nurse vied with her sister in striving to make her room the most attractive of all. The arrangements to enable a nurse to lock up all her possessions, and for making tea at any hour, the ample provision of bathrooms on every floor with a constant hot-water supply, of a large boxroom and excellent lockers for private nurses, with an infinite variety of other details, all supplied to increase the comfort of the staff, must tend to improve their health and promote their happiness. All this must help, too, to raise the standard of efficiency throughout the school.

Nor must it be supposed that the work of a great training school confines itself merely to teaching methods, excellent and necessary though they may be. The welfare of the individual nurse is kept constantly to the fore, and during the fourteen years several developments have taken place in the direction of increasing the nursing staff's facilities for relaxation and exercise, and promoting their general happiness and innocent enjoyment wherever possible. Every probationer has now a fortnight's instead of a week's holiday at the end of each six months, with a final month at the end of her two years' training, and the staff nurses as well as the sisters have four weeks' holiday instead of three weeks as formerly. Each probationer gets a clear day every fortnight and three hours daily off duty for recreation and pleasure. The sisters have every Sunday off in lieu of one Sunday per month, and their half-day has been increased from six to eight hours off duty. In the large wards two sisters are now employed instead of one, so that the duties have been halved. Step by step the hours of the nurses have been reduced or made as little irksome as possible, while the off-duty time has been increased to one day per fortnight instead of one per month in the case of the private nurses, who are now enabled to take seven weeks off duty in the year. The staff nurses and probationers on night duty have now a day and a night off every fortnight instead of every month. The nurses' library has been made a real live thing by constant supervision and the addition of numerous books well and carefully selected. The new nursing homes which have been opened in the period have raised the accommodation for the nursing staff at the London Hospital to a level which many may envy and few surpass. Looking back Miss Lückes may well offer grateful thanks for successful work, and looking forward she may rest assured, when she rests from her labours that her work will follow her.

The purpose of these articles has not been criticism, but it may be helpful to point out, as the result of our knowledge and study of many nurse-training schools a few matters worthy of consideration by those concerned. Has not the time arrived to fall into line with the other training schools, by adopting three years' training and a three years' certificate? Rightly or wrongly this is the general rule practically everywhere, except at the London, and each London nurse has certainly the right, for the purposes of her work after leaving the school, to have a three years' certificate. When is a woman old enough to become a sister? It is no doubt difficult enough to provide for the nursing service of a big hospital like the London, but it is essential in our view to thorough training that every probationer shall have the opportunity of quietly settling down to her new duties, when first moved to a surgical or medical ward, with which object she should remain there for months rather than weeks or days. We feel, too, that the recommendations contained in our article on the need of the training schools, published in our issue of June 9, have a special application in the case of a huge school like that of the London Hospital. We would further suggest that steps should be taken to send two of the best and most capable sisters or matrons' assistants to visit a few of the greater English training schools, and also those of the Massachusetts General Hospital, Boston, the Presbyterian Hospital, and the New York Hospital, N.Y., and the Johns Hopkins Hospital, Baltimore. Such a visit, with the report and information which it should produce, must tend to develop the methods of instruction in various directions, and could not fail to help to perfect the system at the London Hospital. We can quite understand that complete satisfaction is not felt by all the nurses as to the provision for pensions which "will only be payable during the pleasure of the committee." No one would question for a moment the good faith of the present members of the committee, of course, but in the absence of a contract and where the members must necessarily change, committees do not invariably continue the old policy or endorse the views of their predecessors.

### Abdominal Surgery.

By Harold Burrows, M.B., F.R.C.S., Assistant Surgeon to the Seamen's Hospital, Greenwich, and to the Bolingbroke Hospital, Wandsworth Common.

### INTUSSUSCEPTION.

Except in babies, intussusception is very rare, although it may occur at any time of life. The lesion is best explained by the accompanying diagram. Owing to irregular peristalsis or some other cause, a portion of intestine becomes invaginated into the part immediately below.

1. Dilated bowel above the intussusception. 2. Intussuscepted portion of intestine.

This invagination, which frequently has its commencement while the baby is feeding, causes considerable colic, so that the child cries out with pain and refuses to finish the meal.

Now, when an intussusception has taken place, one of two events may come about. Either the invaginated piece of intestine slips out again and a spontaneous cure is established; or, and this must be anticipated as the ordinary outcome of the affair, the invagination increases and more and more of the intestine becomes telescoped into the bowel below.

In the latter case, the circulation of blood in the intussuscepted portion soon becomes arrested, the bowel-wall swells up, and, unless soon released from its imprisonment, becomes gangrenous. In addition to this danger, intestinal obstruction is caused by the swelling of the involved intestine, and babies die even more rapidly than adults from intestinal obstruction. Therefore prompt and active surgical interference is imperative.

To avoid mistakes it is wise to suspect every instance of sudden severe colic in a baby as a possible case of intussusception. If the colic is soon followed by a discharge of blood or blood-stained mucus from the bowel, the presence of an intussusception is almost assured.

The chief danger is to regard the colic as an indication for an aperient and to administer to the wretched baby a dose of castor oil. Such a mistake is deplorable, because castor oil will make the colic worse, will increase peristaltic action, and so cause still more bowel to become invaginated, and will also increase the congestion of the intussuscepted intestine. For these reasons castor oil or other aperient must not be given without careful circumspection to a child with saevere colic.

In a neglected case the child will suffer from persistent vomiting, the abdomen will become distended, and other grave and distressing symptoms will quickly usher in a fatal end.

The aim should be to anticipate these evils by securing proper attention for the child before they arise.

### Treatment.

There are two ways of reducing an intussusception. One is to inject fluid or pump air into the rectum, so as to push back gradually the invaginated portion of bowel into its proper place. The other is to open the abdomen and reduce the intussusception with the fingers. The two methods are sometimes used together, or injection is used first, and if found to fail, laparotomy is performed.

### Inflation.

Inflation, or the distension of the rectum with air, is seldom used now for the reduction of an intussusception, because it is difficult to regulate the pressure of the air which has been introduced. If the pressure is too great there is a danger of bursting the bowel with fatal consequences, and if the pressure is too small the invagination of the intestine will remain. The simplest way of carrying out this method of treatment is by means of a bicycle pump fitted to a catheter. While the inflation is in progress the infant's buttocks must be pressed together so as to stope the air from escaping as fast as it is pumped in.

### Injection.

This is safer and better than the foregoing method. The injection is given as follows: One end of a piece of rubber tubing two feet long is fitted to a glass funnel, and the other end, by means of a glass junction tube, to a rubber catheter. The infant is laid on his back, the catheter introduced well into the rectum, and the funnel held about a foot above the anus. The fluid—warm water, warm normal saline solution, or warm milk—is now poured into the funnel and allowed to flow into the rectum. At the same time the funnel is raised to a height of two feet, and held at this level for a short while. The tubing is then removed and the fluid allowed to flow out of the rectum. The amount that comes away is measured and compared with the amount introduced. The quantities should be practically equal.

The sign that the intussusception has been reduced is the passage of fæcal material. If one attempt fails the safest course is to proceed to laparotomy. But if there are circumstances which render this impossible, a second injection may be given by the rectum, this time the funnel being held at a height of three feet above the anus, and the fluid being allowed to remain in the rectum for a quarter of an hour or more.

The possibility of effecting a cure by these methods—inflation and injection—is due to the fact that the great majority of intussusceptions have their starting-point in the neighbourhood of the ileo-cæcal valve; that is to say, low down in the bowel and within reach of an injection given by the rectum. But it is only in the early stages that either inflation or injection can be used with safety and success. If an intussusception has been in existence for twenty-four hours or more, probably adhesions will exist, and there will be little or no likelihood of reducing the invagination except by laparotomy; moreover, by this time the bowel may have become gangrenous. To attempt a cure by injection in such a case is to waste valuable time and run grave risks for the sake of a negligible chance of success.

### Management of a Case.

When a case of intussusception is admitted to hospital the first duty of the nurse is to obtain from the child's mother as accurate a history of the case as possible. This will include a note as to whether the baby has suffered previously from attacks similar to the present one. The exact time of onset of the colic must be recorded, as well as the interval which elapsed before vomiting, passage of blood by the rectum, and other symptoms ensued. If the infant is being fed artificially a note must be made of the food to which it is accustomed, because it might be unwise to change the character of the food at this critical time. If the child is being fed at the breast, arrangements should be made for this to be continued, and the mother ought to be ready to feed the baby as soon as the operation is over.

When there is vomiting, a specimen of the vomited matter should be preserved, because its character may help the surgeon to form an opinion as to the nature and severity of the case. So also the infant's napkins should be kept so that the surgeon may see for himself the quantity of blood and mucus passed by the bowel.

When the surgeon arrives he will make a rectal examination, he may desire to try the effect of an injection given in the manner described above, and he may find it necessary to perform an immediate operation. Preparations should be made before his arrival for each of these procedures.

With regard to the child, the first precaution of all is the prevention of shock. Towards this end warmth is the great remedy. The baby should be given a warm bath, quickly dried, and wrapped up in a warm blanket. The legs and arms must then be bandaged up in warm cotton-wool. If laparotomy is to be performed, the abdomen should be prepared for operation before the anæsthetic is administered, because it will be desirable to have the period of anæsthesia as brief as possible. A large india-rubber hot-water bottle may be placed under the patient during the operation, if a properly heated table is not available.

After the operation the baby will be transferred at once to a well-warmed cot, placed close to the fire if it be in winter time. As soon as he has recovered sufficiently to be able to suck, he should be fed. Probably this will be within a quarter or half an hour of the operation. This early feeding is most important. The prolonged prohibition of food after anæsthesia, which is the usual practice with adults, is not to be imitated in the case of a baby under treatment for intussusception.

### The Nurses' Clinic.

### THE DISTRICT NURSE AND PNEUMONIA.

In district work the victim to pneumonia is commonly the breadwinner of the family, and as it is a disease in which good or bad nursing may be a matter of life and death, the nurse must go instantly on receiving the summons, and must not leave the patient until all arrangements have been made and the necessary instructions given to the friends.

On arriving at the house the nurse will probably be told that a poultice has already been put on; the ability to make fairly good and hot poultices is very general in all respectable homes—the weak point is in the size of the application, which is rarely more than about 10 in. square. In cases of pneumonia a poultice, to be effectual, must cover the entire lungs back and front; that is to say, must reach from collar bone to the lowest curve of the ribs.

Three or four neighbours, eager to help, will probably have collected in the kitchen, and the nurse should write down the following list of articles, and ask one of the women to go and fetch them : Two pounds of linseed meal (three may be necessary for a very big man), three and a half yards flannelette, three and a half yards white wadding, three and a half yards butter muslin, and a box of medium-sized safety pins. The linseed must not be of the cheap kind, called "horse linseed," which is deficient in oil and will not form an adhesive mass, but freshly crushed linseed, bought from a chemist. The retail price varies from threepence or fourpence a pound to seven pounds for 1s. These quantities of muslin, etc., will suffice to make two full-sized pneumonia jackets.

While these articles are being fetched ask for a large kettle of boiling water, a large tin or enamelled iron basin, an old tea tray, a jug, in which to warm the spatula, and something to make a foundation for the poultice. An old piece of clean sheet can usually be produced; in some instances brown paper is specially ordered by doctors, and they occasionally order the poultice to be tacked between double layers of flannelette. If there is no one to wash and iron the foundations, as in the case of a man in lodgings with no one to do anything for him but a grasping and disobliging landlady, it is often necessary to use brown paper; but as it is an article that is rarely found in poor people's houses, and has to be bought, the convenience is greater than the economy. The nurse must provide the spatula, which should be strong and flexible, the handle six inches long, the blade nine.

As soon as the flannelette, etc., arrive, the nurse should cut out one pneumonia-jacket and set two of the women to work upon it. For a man it should be 25 in. in depth, and folded in such a way before cutting the armholes and slightly curving the neck, that the two fronts together are about four inches wider than the back.

When the jacket is well under weigh, the nurse can begin preparations for the poultice, which should, if possible, be made in the patient's bedroom. If this cannot be done, the poultice, in addition to being made with heated linseed in a hot basin with boiling water, spread with a hot spatula on a warm foundation laid on a hot tray, must be covered with a warm flannel before being carried upstairs. (In theory a jacket-poultice is rolled up, but in practice not one nurse in 500 can make a full-sized poultice that would bear this rather needless test of adhesiveness).

The back poultice should be heavier and wider than the front, and reach some way round the ribs; as soon as it is ready, roll the pneumonia jacket, previously aired and warmed, under the patient; apply the poultice, cover it with

**THE NURSES' CLINIC—*Continued.***

a thick piece of blanket, and fold the pneumonia-jacket round the patient. Make the front poultice and apply it, fastening it closely to the back poultice, well overlapping at the sides and on the shoulders, and cover with a piece of blanket. Always begin at the bottom; three safety pins will be required at each side and two on each shoulder; fasten the pneumonia-jacket in the same way down the front and at the shoulders. If the expense can be borne, a mackintosh should be placed next the poultice. Except in surgical cases, or for exceptionally delicate skins, no muslin is laid between the skin and the linseed meal. For an infant or a very young child the jacket-poultice can be made in one piece. One-lung poultices must always have strings round the neck and waist; pneumonia-jacket as usual.

For an adult one tablespoonful of mustard is generally allowed for the back poultice and one for the front; for children one teaspoonful is sufficient. Sprinkled on the surface (a common practice) it is far too irritating for most skins; the best way is to mix it with the warmed linseed before adding the boiling water.

Poultices are required, as a rule, for twelve days, and they should be changed at least once in six hours, and the pneumonia-jackets dried and worn alternately: the patient's friends must therefore be taught how to make and apply them. When the poultices are discontinued a specially warm pneumonia-jacket must be lined with absorbent wool, and constantly worn.

The patient should lie between blankets, have flannelette pillow-cases, and wear a flannel or flannelette shirt opening all the way down at back or side. A plentiful supply of pillows will be needed, and the only counterpane should be a clean sheet. The room should be kept at a temperature of from 65° F. to 70° F., the necessary moisture being supplied by a boiling kettle. The patient's strength needs keeping up, and the food ordered by the doctor must be given at least every two hours.

Men patients who have been heavy drinkers rarely recover, and as they are usually violent and beyond a woman's power to restrain, the wife should be warned to have a brother or other male relative always at hand.

The nurse should ascertain from the doctor whether the case is septic, as any carelessness in admitting visitors may cost valuable lives.

For some patients camphor-jackets are ordered instead of poultices, and for those who cannot well endure to be moved, or who have insufficient attendance, they are often valuable substitutes. A foundation of lint or white flannelette is soaked in warm camphorated oil and placed in position, covered with jaconet and shaped flannel and a large well-padded pneumonia-jacket. The initial cost is greater, but the whole expense is far less than that of using linseed meal.

The crisis occurs on the eighth day, and in ordinary cases the patient can begin getting up at the end of a fortnight, but ought not to return to outdoor work in less than six weeks from the beginning of the illness. In most cases much time and suffering will ultimately be saved if the patient can be induced at the end of the first month to go to a convalescent home for a fortnight. The pneumonia-jacket should be worn under the ordinary clothes until the lungs have completely recovered their usual condition, and flannel shirts are almost indispensable.

### Incidents in a Nurse's Life.

### A BIRTH IN A TENT.

One cold morning in the early spring a rap came to my bedroom window, which was situated on the ground floor. I thought I might be mistaken, as it was very early—only 3.30 A.M. So I listened, and again came the rap on the window, accompanied this time by a very agitated voice saying, "Nurse, Nurse, are you awake?" With this I jumped out of bed and went over hastily to the window. Without drawing up the blind I said, "Who is there?" The reply came, "Peter." "What Peter?" I again asked. "Peter Mc——. Come quickly, nurse." "Who is ill?" I said. "Ma wife. She's awfu' bad. She took bad at one o'clock this morning. Come quick, nurse." "But where have I to go?" I asked this time, beginning to get a little excited. "Over to D——, nurse." "Oh, yes, Peter, I understand where I have to go now. All right, go you away over, and I will be after you in a few minutes." I started dressing myself in haste, thinking the while of the work before me—a confinement in a gipsy's tent. Lively work, probably under great difficulties. Very soon I was ready, I got out my cycle, and off I went. I soon reached the tent, and putting my cycle against a wall I crossed over and entered—not on my feet, but on my hands and knees, entering in by a hole between some old sacks of which the hut was made. I nearly went in on the top of a fire which was made in the middle, built on the ground in the middle of the tent, and had great difficulties throughout the time from keeping my skirts from being ignited. At one end of the tent I discovered the patient lying on an old mattress and some bracken leaves. After questioning her, I found that baby would not arrive for some time, so, making the necessary arrangements required at such cases and squatting myself tailor-fashion on the ground with a newspaper for a seat, I prepared for a long wait. I was seated between the fire and the patient's "bed," and had to be very careful not to get too near the fire, whilst the smoke went down my throat and into my eyes—the only chimney being a little hole in the roof of the tent, which was scarcely four feet high. While waiting I had a look round. At the other end of the tent were heaps of tin basins, etc., the husband being a tinsmith. Behind the basins and among them lay the rest of Peter's family, four in number. Now and again a little black face—which evidently had not seen water for some months—popped up full of curiosity. I had to give them a threatening look occasionally to keep them from coming to inspect me. At last I asked the father to get them dressed, which did not take long, needless to say, as most of their clothing was slept in, and they were turned out to run about. Glad to get them away, I spoke to the sister-in-law, who, with her husband, occupied the other end of the tent. She was of great use to me later for drawing water, but otherwise was of no assistance, as she was only a young woman of eighteen not long married to the patient's brother. I sat on until it was about 10 A.M. Getting a little hungry and seeing that matters were no further advanced, I said to the patient that I was going home for some breakfast and would be back in a short time. I again mounted my cycle and got home with a face as black as a sweep's from the smoke and soot. After a good wash and tidy-up and something to eat, I again set off for the tent—this time bringing some old pieces of sheets, etc., as there was very little of anything in the tent save tin basins and plenty of water to put in them. As it was nearing twelve noon, I thought I had better send word to the doctor two and a half miles off to come. The patient by this time being much worse, the husband set off, and I again waited. The doctor put in an appearance about 1.30 P.M., and said that baby would not come for at least another two hours, so he went away to make a few calls. About 3 P.M. baby arrived, a great big fat boy, the doctor coming in about five minutes after. We soon got everything put right about the patient, and the doctor quitted the tent, leaving me to see to the baby. I soon got it washed and dressed with what I could find, and put it as cosily as possible beside the mother. Tidying myself a little and shaking out my clothing on my knees—as it was quite impossible to stand straight up without putting one's head out at the chimney, which would be anything but pleasant—I said I should look in in the evening, gave instructions, and departed as best I could on my cycle. I found myself terribly sore and tired owing to my cramped position in the tent, not having been able to stand up straight from 3.45 A.M. till 4 P.M. Needless to say, I had more than smoke to make me dirty and uncomfortable during my stay, but as soon as I reached home I shook all I found on my clothes with quick despatch into some carbolic 1.20, which I have no doubt speedily finished their career.

### The Evolution of a Poor-Law Training School.

AN INTERVIEW WITH THE MATRON OF ISLEWORTH INFIRMARY. BY OUR COMMISSIONER.

VISITING Isleworth the other day in order to have a chat with the matron of Brentford Union Infirmary, I made a mistake which it appears is far from being without precedent. The very handsome new workhouse, with a church of considerable size, is in proximity to the infirmary, and, asking for the matron at the entrance, I was conducted into the presence of a lady who evidently did not expect me. An explanation followed, and I was courteously directed to the fine block of buildings some distance further, with a frontage to the main road. It is only ten years since the infirmary was opened, and yet important extensions are now being made. These, as Miss Moriarty, the matron, informed me, have, however, become absolutely necessary, owing to the steady increase in the number of patients. Proceeding through several of the wards, which are lofty, well-appointed, bright with summer flowers, and have the advantage of open corridors connecting them, the matron told me, in effect, the story of the evolution of a training school.

### The Start.

"It began when this infirmary was opened?" I asked.

"Yes, when I took up my duties on my appointment as first matron just about ten years ago."

"You had enjoyed experience in Poor-law nursing?"

"For eight years I was Home Sister at St. Marylebone Infirmary under Miss Vincent; but I was trained at St. Thomas's Hospital, where I was awarded the three Nightingale letters and gratuities."

"Of course, you started here with a small staff?"

"We commenced with a staff of twelve, five of whom were probationers. The period of training has from the outset been three years, each probationer being required at the end of two months' trial to sign an agreement, in which she states that, having become practically acquainted with the duties of a probationer in the infirmary, she is satisfied that she shall be able and willing to enter into service, engages to abide by the regulations, and to continue in the employment of the Guardians for the three years."

"Are there any special features in the regulations?"

"I do not think so. The age considered desirable for probationers is between twenty-two and thirty; the salary for the first year is £5, the second £10, and the third £20. As to theoretical training, lectures are given by the medical officers, the classes are held by me and my assistant, and every opportunity is afforded the probationers of assisting in the operating theatre, in order that they may obtain a thorough knowledge of surgical work. The certificate, which is recognised by all nursing authorities and by the Local Government Board, is given after a nurse has passed her final examination, which is held by an outside examiner."

### Massage and Midwifery.

"I see that in your regulations you mention that training is given in massage?"

"There are regular classes, taken by a sister, who also instructs in practical work. The classes start in September, to enable the nurses to go in for the February examination of the Incorporated Society of Trained Masseuses. If I spare them to attend the classes, I expect them to enter for the examination, and, as a matter of fact, during the six years which have elapsed since the classes were started thirty-four of our nurses have received the certificate of the Society."

"You attach a good deal of importance to training in massage?"

"I am quite sure that it is very important. One of our nurses who did not take the course deeply regretted it afterwards; and, on the other hand, an Isleworth nurse, who took the course, became massage sister at the Birmingham General Hospital. In private nursing it will become, if it has not already become, indispensable."

"What are your arrangements as to midwifery training?"

"Nurses who wish to have it give their services free for six months after they have received their certificate for general training. I have two sisters and one nurse here who have taken the course; and during three years ten pupils have either passed the examination of the London Obstetrical Society or the Central Midwives Board, there not being one failure."

"The three years to which you refer mean, I conclude, the period which has elapsed since the maternity ward was opened?"

"Yes; it was then transferred from the workhouse, sixteen beds being provided for the lying-in. The staff consists of a sister-midwife and two pupils.

### The Existing Staff.

"Has your progress in numbers during the ten years been gradual?"

"No, we had our full staff very soon. It now consists of eight sisters, one night sister, and twenty-five nurses in different stages of probation. These figures include the staff in the maternity wards. As to the number of beds, we started with 88, and there are now 320, which will be considerably augmented when the two pavilions now in course of construction are completed. I expect they will be open in about nine months."

"How are the wards staffed?"

"On the female side, for the flat consisting of two wards, there are on duty a sister, a day and night staff nurse, and two probationers; on the male side, a sister, a day and night nurse, and one probationer; and in the children's ward an extra probationer on night duty. Generally speaking, the probationers have very little night duty in their first year, but when they are promoted in their second year to be staff nurses they take a stretch of five months. The majority like night duty."

"What about their hours?"

### THE EVOLUTION OF A POOR-LAW TRAINING SCHOOL—*continued.*

"They rise at 7 P.M., are in the wards at 8.30, leave them at 8.30 A.M., dine at 9 A.M., have exercise from 9.30 to 11.30 A.M., and are in bed by 12 noon. They are off duty on Sunday, cases permitting, from 10 A.M. to 1 P.M. The day nurses have from 10 A.M. to 1 P.M., from 2 P.M. to 5 P.M., or from 6 P.M. to 10 P.M. The nurses on day duty rise at 5.45 A.M., breakfast at 6.30 A.M., are in the wards at 7, have lunch at 9.30 to 10, dinner from 1 to 1.30 P.M., tea 5 to 5.30 P.M., are off duty at 8.30 P.M., have

The Nurses of Isleworth Infirmary.

supper at 9, and are in their bedroom at 10 P.M. Each probationer has exercise from 10 to 12 A.M. or 2 to 4 P.M., staff nurses 2 P.M. to 4 P.M. or 6.30 P.M. to 9 P.M. or 10 P.M., and a day off once a month as well as three weeks' holiday at the end of the year."

### The Nurses' Home.

"I see that meals are served in the infirmary."

"Yes, in the dining-room, but the nurses "off duty" are allowed to have supper in the home. The home has accommodation for thirty-three nurses, each having a separate bedroom, and, as you noticed, a fireplace. Each room is provided with cupboards, which save space and are convenient. The recreation-room contains a piano and an ample supply of easy-chairs. The classes are held in the study, and there is a visitors' room, in which nurses can see their friends and give them tea."

"Who presides at the meals in the infirmary?"

"The assistant matron, and if any of the staff do not take their food they are not allowed to work. Their health is always carefully looked after, and for six months last year I had not a single nurse off duty for one day owing to indisposition."

### The Interest of the Authorities.

"You have nice lawns for tennis and croquet."

"The garden is much appreciated; in fact, taking things altogether, I am afraid that in such institutions as this there is a danger of being spoilt for private life."

"Here you have, evidently, no cause of complaint against the authorities."

"On the contrary, the Infirmary Committee of the Brentford Board of Guardians have always been ready to give me every assistance, and they are very kind to the nurses. To the late medical superintendent, Dr. Walter Fooks, who was here for nearly eight years and helped so materially in the formation of the training school, and to the present, Dr. Everett Norton, I am indebted for their most cordial co-operation. Indeed, I have never had any difficulty."

"Do you have many infirm patients?"

Only those needing nursing care. The infirm wards are in the workhouse. There are a great many patients treated in the operating-room. We had a case brought in three days ago of attempted suicide, shot through the chest. The bullet was extracted and the man is doing well. The trams supply us with many adult accident cases, and the district schools with juveniles. One boy has broken his arm three times. Then there are three wards for pthisical cases—two for men and one for women—with the outside treatment. It is an immense relief to some of the patients to be outside, but others prefer to remain in the wards. The windows are always open."

### The Isleworth Graduates.

"Can you tell me how many nurses have now received your certificate of training?"

"There were fifty-one on the 8th of this month. You may like to know that of these seventeen are engaged in private nursing, five are sisters and four staff nurses here, two matrons of cottage hospitals, one sister in the Royal Navy, one a superintendent Queen's nurse, one a district nurse, three sisters in hospitals, three sisters in Poor-law infirmaries, and six more left Isleworth in order to become sisters in hospitals."

"With regard to private nursing, do you think that probationers trained in Poor-law schools are particularly well qualified for it?"

"Speaking as a hospital-trained nurse myself, I am sure that they are better suited for private work than hospital-trained nurses. As probationers they are obliged to be sympathetic and kindly to individual patients. For a rush of work in a great institution, hospital-trained nurses are better."

"Do you get plenty of applications?"

"More than enough of a kind. The difficulty is to get them of the right stamp—to secure probationers who combine both capacity and education. Well-educated girls coming into the Poor-law infirmaries can do useful work. The higher posts are open to them. Infirmary graduates

The Nurses' Recreation-room at Isleworth Infirmary.

are best suited for the higher posts in infirmaries. I should not care to have anyone as assistant matron who came straight from a hospital. If it had not been for my experience at St. Marylebone Infirmary I should never have been able to do the work here."

Children's Training and Registration.

"Have you any objection to probationers who have already had training at a children's hospital?"

"It depends considerably upon where they have received it. But as a rule I prefer that they should not have had training in any other institution. A girl can learn punctuality, forethought, and discipline as a nursery governess, but I think that it is better for her not to learn a branch of nursing before she enters an institution for general training."

"Are you in favour of State registration?"

"I do not see the least use of it to anyone. Incapable nurses often pass an examination easily, and no certificate can be a guarantee of character. I believe in nurses who regard nursing as a vocation, and do not look at it from the 'case' point of view."

### Moving a Patient Suffering from Heart Disease and Dropsy.

### EXAMINATION QUESTIONS FOR NURSES.

The question was as follows: What arrangements, in a poor cottage, would you make for a patient who is heavy and suffering from heart disease and dropsy, to aid in the moving that is so much desired by those suffering from these maladies?

### First Prize.

In a poor cottage, with a heavy patient suffering from heart disease and dropsy, a rope fastened securely to a ring in the ceiling over patient's bed would assist greatly in lifting. We must have a good strong draw-sheet, which we know is so useful in lifting helpless ones. Some patients are much relieved by a forward rest; this could be made by two perpendicular pieces of wood a foot wide, and a little higher than the bed, connected together by a plank a little longer than the bed is wide This, with a pillow, affords comfort for the arms. A back-rest could be substituted by a chair turned upside down and made comfortable by pillows. To prevent slipping down the bed, a roller pillow put under the buttocks and tied firmly with strong tape to the head of the bed, or a firm bolster put across the bottom of the bed would partially help.—"Poor Pug."

### Second Prize.

In cases of heavy patients suffering from heart disease and dropsy, one is aware how continually they crave attention, as there is always a tendency with heavy patients to slip down the bed a few minutes after being comfortably propped up. Bed-rests and blocks are things unknown; but possibly some rich neighbour will lend an air-cushion or water-pillow. Fetch from the kitchen an ordinary wooden chair, also a round roller towel, a couple of bricks or a few books, and an old soap-box. Neighbours are always kind enough to call and see or condole with anyone sick of their own grade, so now we will ask the kindly assistance. Invert the chair at the head of the bed, the two front legs of it resting on bed-head; pack with pillows laid lengthwise; put one arm behind the patient's shoulder, and the other hand top corner of the draw-sheet, the neighbour doing the same; give your patient one end of the roller towel, after fastening the other to rail at the bottom of bed, just to keep her hands and head employed, not to exert herself by pulling, then lift. Pass a firm pillow under the knees, covered, of course, by some homely protective. Slip the soap-box under the mattress and put the books under the bedstead legs at the foot of the bed. Leave the towel attached to the bed-foot, as it may be slipped over the patient's head and shoulders whilst the pillows are shaken up. A few oat-chaff cushions made small can be inserted to minimise any discomfort arising from crevices, or help to change of position without exertion, which is so bad for the patient.—"Spring."

### The Prize-winners.

The papers attain a better standard this month, though some good answers are spoilt by being far too diffuse, and some others by neglecting the fact that patients suffering from heart disease and dropsy are almost always extremely helpless. Both the prize papers are good, and "Spring" would have gained the first, because her description of the manner of doing the actual lifting is excellent, but she omitted the important matter of tying the bolster placed under the thighs to the head of the bed. "Poor Pug" remembers this, and also has a good plan for giving a forward resting-place, a manœuvre most useful in heart cases.

### Honourable Mention.

This is gained by "Few," "Nil Desperandum," "Queenie," and "Scottie." Another candidate would have gained this distinction, but she sent her paper without name or address!

### No further Competitions until October.

There will be no further questions until October, when most nurses will have returned from their holidays.

The Examiner.

### Rules.

The competition is open to all. Answers must not exceed 500 words, and must be written on one side of the paper only, without divisions, head lines, or marginal notes. The pseudonym, as well as the proper name and address, must be written on the *same* paper, and *not on a separate sheet.* Papers may be sent in for 15 days only from the day of the publication of the question. All illustrations strictly prohibited. Failure to comply with these rules will disqualify the candidate for competition. Prizes will be awarded for the best two answers. Papers to be sent to "The Editor," with "Examination" written on the left-hand corner of the envelope.

In addition to two prizes honourable mention cards will be awarded to those who have sent in exceptionally good papers.

N.B.—The decision of the Examiner is final, and no correspondence on the subject can be entertained.

Any competitor having gained three prizes within the current year shall be disqualified from taking another until 12 months shall have expired since the first prize was gained.

### The Management of Children.

The 650 pages of Dr. Barrett's book * form a very complete handbook to the management of children in health and in disease, from birth to the age of puberty. Conscious that the book, especially perhaps the American edition of it, will come into the hands of both mothers and nurses who are far from medical assistance in the hour of need, the author gives a good deal of advice as to treatment, though he recommends the calling in of medical assistance in sickness or other emergencies wherever possible. If one could be sure of always having a doctor at hand, there would be little need for such elaborate treatises as the one under consideration, for no book, especially when it is read and construed by a lay person, can give such efficient guidance as an experienced medical man, and one cannot but be conscious that as much harm is often done by the mother who flies to her "Home Doctor" or "Mother's Adviser" and drugs and blisters according to her untaught opinions, as by the one who trusts too entirely to the *vis medicatrix naturæ.* It is rather strange, considering how tender a creature a child is, and how precious to its parents, that these parents often show themselves so thoughtless in regard to its welfare.

* "The Management of Children: A Book for Mothers and Nurses." By Howard Barrett, Member of the Royal College of Surgeons of England, Fellow of the Royal Medical and Chirurgical Society, Fellow of the Medical Society of London, late Surgeon to Poplar Hospital, etc. Illustrated. (London: George Routledge and Sons, Limited, Broadway House, Ludgate Hill. New York: E. P. Dutton and Co. 5s. net.)

The thoughtlessness is often due to ignorance. There is a notion prevalent that mothers have an instinctive knowledge of the needs of children, and can bring them up without assistance or advice from outsiders. Sentimentalists pretend that love brings knowledge, but it is the most affectionate mothers who are most conscious of their ignorance, and long the most for experienced help. Many women seem to think that a child will grow up with as little care as a kitten. They do not devote their own time to their babies—finding social or other duties more importunate—nor do they take much trouble to obtain an experienced nurse who might be as good, or, thanks to her experience, even better than a young mother. Even rich people seem often to grudge the wages of a really good nurse, while the lower down one goes into the servant-keeping class, the more inexperienced and rough the nurse seems to be. It must shock every true lover of children to see, as one so often does, a slatternly ex-schoolgirl—too frequently dressed out in a travesty of a hospital garb—gossiping with a friend of the same class at a cold street corner, while her charge looks blue and miserable, or perhaps has fallen asleep in some uncomfortable posture. People who insist on a good cook and a clean housemaid seem to think that anything in the way of leggy girlhood will do to look after a baby. There is a comprehensible preference for young nurses, because they are more willing to play with a child, and where the mother is experienced and is willing to devote the greater part of her time to her child, such a girl may be useful enough, but a young mother and a young nurse make a bad combination, and it would be well if mothers would realise that the wages of a good nurse will be saved again and again in doctor's bills in the near and more distant future.

But it must be confessed that nurses themselves—we mean good nurses—are less willing than they might be to take charge of children. Thanks in great part to the leggy slatterns of whom we have spoken, the nurse to healthy children does not receive as high social consideration as that of the sick-nurse. The more is the pity, and the more we respect those mothers—alas, too few!—who seek as the companion and caretaker of their children a trained, capable, refined, and educated woman, and treat her as she ought to be treated. They have their own immediate reward, and we trust that in time they will set a wholesome fashion in the nursery world. But if the fashion is ever to become general, the nurses must be ready for it. A gentlewoman who has gone through a proper, complete training in a children's hospital should not think it beneath her to take a post as children's nurse—always presupposing that the salary and the consideration are such as her competence deserves. For many ladies the post would be more satisfactory than that of the very indefinite "nursery governess," and it is less fatiguing than sick-nursing. In many cases it would provide the nurse with a home where she would be loved and honoured, and from which, if she chose to remain, it is unlikely that she would be expelled even after her charges outgrew the nursery stage.

### Wants and Workers.

The matron of the Littlehampton Cottage Hospital will be very grateful if any lady having no further use for her mailcart or perambulator will be good enough to send it to her for the patients.

### Where to Go.

Sale of Work at 76 Addison Road, Kensington.—On Thursday, July 5, at 3 p.m., in aid of the Central London Throat and Ear Hospital, Gray's Inn Road.

Sale of Work, North London Nursing Association Home, 413 Holloway Road, N.—July 3rd and 4th, 2.30. Free after 6 p.m. on July 4th.

### Grand Bazaar at the Albert Hall in aid of Great Northern Central Hospital.

On Tuesday last the above bazaar was opened by H.R.H. Princess Christian, who was conducted to the platform by Sir John Dickson Poynder, the chairman of the hospital. In his address Sir John made two interesting statements. In the first place he based his appeal on the fact that, notwithstanding the average deficit of £600 per annum, which accumulates as time goes on, no modern hospital is complete without a convalescent home, and it is partly to provide this that funds are required. Secondly, Sir John Poynder went on to say that no hospital in London could be regarded as a local institution; all Londoners, he urged, are bound to loyally support such hospitals as exist in the Metropolis, and thus his West End audience should feel a personal interest in the great hospital of North London.

With the stimulus given by H.R.H. Princess Christian's presence the bazaar went merrily, and indeed the stalls surrounding the arena and the guests moving between them made a very pretty scene. The decorations were charming, and it would be difficult to say which stall was actually the best done.

Books, hats, jewellery, sweets, and many other articles were represented by stalls, and we were delighted to see some of the hospital nurses with a stall of their own. During the whole afternoon a selection of music was played by Vorzanger's Blue Austrian Band.

It may interest our readers to be reminded that the Great Northern Central Hospital possesses wards for paying patients, and thus is very largely free from the charge now so constantly brought against hospitals of admitting patients who can afford to pay free of charge. In these days, when the cry of extravagant management is being everywhere heard, a hospital which offers special facilities for paying patients is one that is specially deserving of support. We can only hope that this bazaar at the Albert Hall will realise the best hopes of its promoters, and we can guarantee that an afternoon spent first at the bazaar and concluding with an admirable concert made an exceedingly good entertainment for all who had the good fortune to attend.

### Everybody's Opinion.

### "THE PRESCRIBING NURSE WE KNOW, BUT WHAT IS THIS"?

"Surgeon" writes from the British Hospital, Nazareth, Palestine, June 3rd, 1906:—Our English qualified nurse receives your paper regularly, and occasionally a copy falls into my hands. By chance I was glancing over the issue for February 3rd, when I noticed the article on page 275 entitled "District Nursing in East London." It contains much that is interesting; but one sentence at least seems to me curiously foreign to a journal which I have always understood to stand for all that is "correct" in the nursing profession. The "Nurse" who writes the article, in speaking of the case of ulcer of the ankle, explains how difficult it was to heal "until I had grafted some of my own skin on to it." The prescribing nurse we know—may her number decrease!—but what is this?

### DUTY AND DISCIPLINE.

M. Barter and A. Stredwick write: We regret to notice in your issue of June 23 a very inaccurate version of the recent occurrence at Kingston Union Infirmary, and as two of the sisters concerned, trained in the institution, and having an aggregate of more than nine years' service, we feel bound in justice to ourselves and our friends to offer a strong protest. Your article states: "It is well known that the relations existing between the matron and her staff have always been particularly happy"; whereas it is perfectly well known that at no time in the history of the institution has this been the case. Again, no special instructions such as you mention had been given, and there was no patient on either of the three floors who was at that particular time nearing the point of death. As to the preparing of tea, it was made by the sister of the surgical floor and handed to the others on the stairs, so that they were only away from their wards a fraction of a minute. Moreover, it had become an established custom to drink a cup of tea after luncheon, since the privilege of making it in the home, which we formerly enjoyed, was for some unexplained reason taken away from us. We must also state that we have frequently had to leave the wards on other occasions, such as to notify to matron the presence of a guardian, or to take down a sick note to the office, and it would have constituted an offence if we had omitted to do either. We expressed at the time our regret to matron and were perfectly willing to promise that no tea should in future be prepared, but we strongly resented the suggestion that we should resign before committee met, and on our ignoring that, being ordered, as a punishment and in a very peremptory manner, to give up the charge of our wards to which we had become much attached. We preferred to take our punishment from the hands of the committee instead, and as we had tendered all the apology we could honestly offer at the time of the occurrence we chose to take the alternative they offered us and resign. Trusting to your courtesy and fair dealing to give this letter the publicity we desire.

### ARE NURSES UNDERPAID?

"Common Sense" writes:—Being a Queen's Nurse, I should like to thank Mr. Pollitt for his interest in district nurses, and trust that steps will soon be taken to bring his suggestions into effect. Mr. Pollitt states that the difficulty experienced by a nurse in saving with a salary of £40 a year. He does not think that sum sufficient to enable one to make provision for that period in which work is no longer possible. Yet there are many nurses who never rise to that amount, few, comparatively, exceeding £35 a year. True, it can be obtained in Blackburn after some years' service; but in many of the Homes the salary of staff nurses does not exceed £35, and in single posts the condition is similar. There is a point affecting nurses in which a little "educating" of the committees in the various districts would result advantageously to the nurses, and would not be at all difficult to grasp, be the committees ever so obtuse. A nurse, after two years' service to the Institute, usually receives £35 a year; if she changes her district, then or later, she loses, having to begin again at £30. Now the point is this: if that nurse fills the vacancy of one who has been receiving £35 a year, should she not get it too? She has done her two years' work, and gained experience in another place, and any committee understanding that the nurse to be appointed is as experienced as the one leaving would not offer a less salary. It is a pity that the time already served cannot be more recognised in making new appointments. Thus, instead of reverting again to £30, as is often the case, the nurse would receive at least that amount she had been having in the district she vacated. Then what a help to the nurses if the Jubilee Institute could devise a means of assisting them in paying premiums to the Pension Fund. Such a scheme would make it possible to provide for the future, and would induce many to serve the Institute for longer periods, and so contributing to the curtailment of expenditure in training fresh batches of nurses. Many Queen's Nurses love their work. To them there can be "no better or holier calling"—as expressed by Queen Alexandra in her speech addressed to them at the reception at Marlborough House a few years ago. When these nurses retreat from the field for private or other lucrative work, it is because they recognise the necessity of making hay while the sun shines, or, in other words, making provision, while they can, for the inevitable winter of life. Reading the report of the Junius S. Morgan Benevolent Fund, I was struck by the articles provided by the Fund for three nurses, as well as the Christmas gifts of money and warm garments to the annuitants. The report speaks for itself, and should awaken nurses in time to consider their position for the future, and take steps, as far as possible, to ensure a latter end of comfort and independence. District nurses, and those whose work lies among the masses, see the necessity of saving for the rainy day. They witness daily the straits of the poor and their struggles for existence. The Scottish branch of the Jubilee Institute has adopted a pension scheme for its nurses. Why cannot England follow and assist its poorer sister—Ireland?

### Appointments.

Hospital for Women, Shaw Street, Liverpool.—Miss Annie L. Wilson has been appointed lady superintendent. She was trained at the Royal Infirmary, Aberdeen, where she has since been assistant matron and home sister.

Leeds General Infirmary.—Miss Harriet Deakin has been appointed assistant superintendent. She was trained at the General Infirmary, Leeds, where she has since been sister in charge of the casualty and out-patients' department and sister in charge of a men's ward. She has subsequently been night superintendent at the Infectious Hospital, Newcastle-on-Tyne, and assistant matron at Bradford Royal Infirmary.

North Eastern Fever Hospital, Tottenham.—Miss Lily Beatrice Maud Hall has been appointed charge nurse. She was trained at Whipps Cross Infirmary, Leytonstone. She has since had fever training at the North Western and South Eastern Hospitals under the Metropolitan Asylums Board. She has also done private nursing and has been nurse at the Hampstead Infirmary.

Royal South Hants and Southampton Hospital.—Miss Mary Carruthers has been appointed holiday night superintendent. She was trained at the General Infirmary, Huddersfield, and has since been sister at West Ham and East London Hospital; sister at the National Hospital, Bloomsbury; and night sister at Torbay Hospital, Torquay.

Southampton Isolation Hospital.—Miss Kathleen Murphy has been appointed charge nurse. She was trained at the Mater Misericordiæ Hospital, Dublin, and has since been charge nurse at the Park Hospital, Hither Green, S.E., and the Fountain Hospital, Tooting, S.W. She has also done private nursing for two years.

### Presentations.

Bedworth and District Nursing Association.—Nurse Holmes, who for nine years was district nurse under the Bedworth Original Nursing Association, has been presented with a silver tea-service, in recognition of the work she has done in the parish. The presentation was made by Mr. Johnson, M.P.

### Death in our Ranks.

The death of Miss Nellie Cotton, who during the year she was probationer at Birmingham General Hospital endeared herself to those associated with her, took place on Friday last, after a long illness, at the house of her sister in Coventry.

### Novelties for Nurses.

(By our Shopping Correspondent.)

### "4711" EAU DE COLOGNE.

At this season of the year a bottle of eau de Cologne, with its refreshing and beautifying perfume, is an extremely acceptable gift. Moreover, as nurses know, when it is made of ingredients of the best description, such as those employed in the manufacture of 4711 eau de Cologne, it is also no less valuable as an antiseptic in the sick room than as a restorative in the case of headache. As an adjunct to the toilet it is most delightful.

### Notes and Queries.

**REGULATIONS.**

**The Editor is always willing to answer in this column, without any fee, all reasonable questions, as soon as possible.**

**But the following rules must be carefully observed.**

**1. Every communication must be accompanied by the name and address of the writer.**

**2. The question must always bear upon nursing, directly or indirectly.**

**If an answer is required by letter a fee of half-a-crown must be enclosed with the note containing the inquiry.**

*Two Years' Waiting.*

(162) I wish to become a nurse in two years' time, but 18 months ago I underwent an operation for hernia. I am quite strong now. Am I likely to be able to enter a hospital later? If so, please tell me what books to read in the meantime.—*Pembroke Dock.*

If you are really quite strong there is no reason why you should not enter a hospital in two years' time. By all means study physiology, anatomy, and sick-room cookery. Write to The Scientific Press, 28 Southampton Street, Strand, for their catalogue of manuals.

*Text Books.*

(163) Can you advise me what books to read before entering the Nightingale Home?—*Wood Bee.*

It would be better to write to the Lady Superintendent and ask for particulars.

*Training School.*

(164) Is the North Staffordshire Infirmary a recognised training school, and is there a medical school attached?—*Inquirer.*

There is no medical school attached to this infirmary, but it has 216 beds, and gives a recognised three years' training.

*Under Twenty-three.*

(165) Can you tell me if there is any general hospital where probationers are received under 23?—*Doris.*

We know of no such general hospital.

*Diet.*

(166) Can you give me the address of a diet specialist in London and the addresses of two eminent medical men whose names I enclose?—*E. G.*

We cannot recommend individuals. The addresses of the two medical men you name will be found in the Medical Directory, copy of which can be consulted at many free libraries.

*Electricity.*

(167) Can you tell me where to take lessons in medical electricity?—*Masseuse.*

We cannot give the name of a private establishment, but you might write to the General Hospital, and ask if you could study there.

*Home for Epileptic.*

(168) Can you help me to find a home for partly paralysed man? Can pay a small sum.—*H. W. W.*

Not knowing all particulars, we cannot recommend any individual home. But you will find a list of homes and charities, which may help you, in Burdett's "Hospitals and Charities," to be obtained from the Scientific Press, 28 Southampton Street, Strand, W.C.

*Religious Homes.*

(169) Where can I get training under religious sisters at Gloucester?—*Mary.*

At St. Lucy's Hospital, Kingsholm, Gloucester, which is conducted by the Clewer Sisters.

*Rheumatoid Arthritis.*

(170) Can you tell me of an institution successful in the treatment of arthritis?

The Buxton Bath Charity, or the Devonshire Hospital at Pulton, is a very suitable institution.

*Consumption.*

(171) Can you help me to find a home for an incurable case of phthisis in a woman, and one that she can be admitted to immediately?—*E. C.*

Unfortunately, we know of no home where she could be certain of immediate admission. There is a home for consumptive females at 58 Gloucester Place, Portman Square, London. Also one for advanced cases at St. Catharine's Home, Grove Road, Ventnor, and at St. Barnabas Home, Brockett Hall, Torquay.

**Handbooks for Nurses.**

| | Post Free. |
|---|---|
| "How to Become a Nurse: How and Where to Train." | 2s. 4d. |
| "Nursing: its Theory and Practice." (Lewis.) ... | 3s. 6d. |
| "Nurses' Pronouncing Dictionary of Medical Terms." | 2s. 6d. |
| "Complete Handbook of Midwifery." (Watson.) ... | 6s. 4d. |
| "Preparation for Operation in Private Houses." ... | 0s. 6d. |

**Of all booksellers or of The Scientific Press, Limited, 28 & 29 Southampton Street, Strand, London, W.C.**

### For Reading to the Sick.

IN THY HAND.

I thank Thee I am not mine own,
But have to live in Thee alone,
Each passing day, each passing hour,
To live in Thy great power.
Whate'er to-day, to-morrow brings,
'Tis all Thine hand, Thine orderings.

*Isaac Williams.*

Nothing in life has any meaning, except as it draws us further into God and presses us more closely to Him. The world is no better than a complication of awkward riddles, or a gloomy storehouse of disquieting mysteries, unless we look at it by the light of this simple truth, that the eternal God is blessedly the last and only end of every soul of man.—*F. W. Faber.*

In a life united with Jesus Christ and in that life alone is the happiness of humanity; for there alone is the true sphere of human life found. All lives that own any other centre than Jesus are swallowed up with hunger and with thirst. Over every other well, to which thirsting humanity flocks to slake its agonising thirst, the experience of the generations of men has written: "He that drinketh of this water shall thirst again," but they who drink of the water of life never thirst. In Jesus Christ is the satisfaction of every want, and the quieting of every fear, for in the life of the justified on earth is the anticipation of the life of the glorified in heaven—even of that full development of the varied powers of our being, and that perfect satisfaction which awaits the redeemed of God in the heavenly court, "where they hunger no more, nor thirst any more, for the Lamb Who is in the midst of the throne does feed them."

*Canon Body.*

Be not anxious about little things if thou wouldst learn to trust God with thine all. Act upon faith in little things; commit thy daily cares and anxieties to Him, and He will strengthen thy faith for greater trials that may come. Rather, give thy whole self into God's hands, and so trust Him to take care of thee in all lesser things, as being His, for His own sake, Whose thou art.—*E. B. P.*

Grant to me above all things that can be desired, to rest in Thee, and in Thee to have my heart at peace. Thou art the true Peace of the heart; out of Thee all things are hard and restless. In this very Peace, that is, in Thee, the One Chiefest Eternal Good, I will sleep and rest.—*Thomas à Kempis.*

As Christ upon the Cross
In death reclined,
Into His Father's Hands
His parting soul resigned;
So now herself my soul
Would wholly give
Into His sacred charge
In whom all spirits live:

So now beneath His Eye
Would calmly rest,
Without a wish or thought
Abiding in the breast,
Save that His will be done.

*Latin Hymn.*

## Figures and Tables

**Figure f1:**
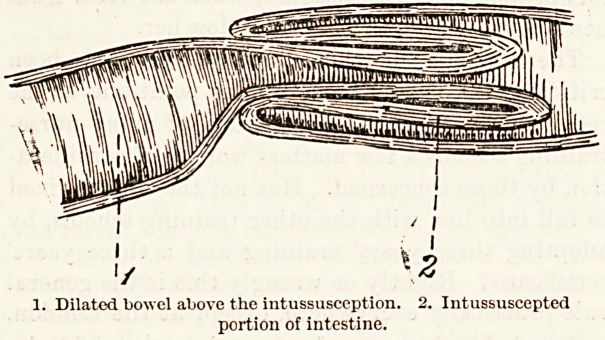


**Figure f2:**
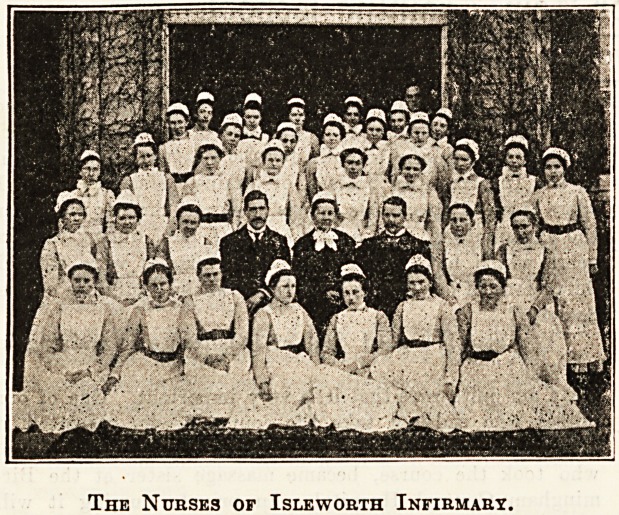


**Figure f3:**